# Specific and Sensitive Detection of Neuroblastoma mRNA Markers by Multiplex RT-qPCR

**DOI:** 10.3390/cancers13010150

**Published:** 2021-01-05

**Authors:** Lieke M. J. van Zogchel, Lily Zappeij-Kannegieter, Ahmad Javadi, Marjolein Lugtigheid, Nina U. Gelineau, Nathalie S. M. Lak, Danny A. Zwijnenburg, Jan Koster, Janine Stutterheim, C. Ellen van der Schoot, Godelieve A. M. Tytgat

**Affiliations:** 1 Princess Maxima Center for Pediatric Oncology, Department of Pediatric Oncology, 3584 CS Utrecht, The Netherlands; L.M.J.vanZogchel@prinsesmaximacentrum.nl (L.M.J.v.Z.); n.u.gelineau@prinsesmaximacentrum.nl (N.U.G.); n.s.m.lak-4@prinsesmaximacentrum.nl (N.S.M.L.); j.stutterheim@prinsesmaximacentrum.nl (J.S.); 2 Sanquin Research and Landsteiner Laboratory, Department of Experimental Immunohematology, Amsterdam UMC, University of Amsterdam, 1066 CX Amsterdam, The Netherlands; l.zappeij@sanquin.nl (L.Z.-K.); a.javadi@sanquin.nl (A.J.); marjoleinlugtigheid@gmail.com (M.L.); e.vanderschoot@sanquin.nl (C.E.v.d.S.); 3 Academic Medical Center, Department of Oncogenomics, 1105 AZ Amsterdam, The Netherlands; d.a.zwijnenburg@amsterdamumc.nl (D.A.Z.); jankoster@amsterdamumc.nl (J.K.)

**Keywords:** neuroblastoma, minimal residual disease, RT-qPCR, metastasis

## Abstract

**Simple Summary:**

Sensitive detection of minimal residual disease by RT-qPCR in patients with neuroblastoma is shown to be predictive of outcome, but has not yet been introduced into clinical practice. A panel of multiple mRNA markers increases the sensitivity of minimal residual disease detection, since neuroblastoma tumors are heterogeneous tumors. Recent studies have identified two distinct phenotypes, an adrenergic and mesenchymal phenotype, that can be identified by using different mRNA markers. As generally only small volumes of bone marrow or blood are available in young neuroblastoma patients, we have developed a multiplex RT-qPCR to be able to test seven different mRNA markers, while we reduce the sample volume needed. Comparison between the multiplex RT-qPCR and RT-qPCR for the single markers showed a comparable sensitivity. This reduction in required sample volume, while saving time and resources, can assist in the introduction of minimal residual disease detection by RT-qPCR into clinical practice.

**Abstract:**

mRNA RT-qPCR is shown to be a very sensitive technique to detect minimal residual disease (MRD) in patients with neuroblastoma. Multiple mRNA markers are known to detect heterogeneous neuroblastoma cells in bone marrow (BM) or blood from patients. However, the limited volumes of BM and blood available can hamper the detection of multiple markers. To make optimal use of these samples, we developed a multiplex RT-qPCR for the detection of MRD in neuroblastoma. *GUSB* and *PHOX2B* were tested as single markers. The adrenergic markers *TH*, *GAP43*, *CHRNA3* and *DBH* and mesenchymal markers *POSTN*, *PRRX1* and *FMO3* were tested in multiplex. Using control blood and BM, we established new thresholds for positivity. Comparison of multiplex and singleplex RT-qPCR results from 21 blood and 24 BM samples from neuroblastoma patients demonstrated a comparable sensitivity. With this multiplex RT-qPCR, we are able to test seven different neuroblastoma mRNA markers, which overcomes tumor heterogeneity and improves sensitivity of MRD detection, even in those samples of low RNA quantity. With resources and time being saved, reduction in sample volume and consumables can assist in the introduction of MRD by RT-qPCR into clinical practice.

## 1. Introduction

Disseminated disease to the bone marrow (BM) is present at diagnosis in more than half of the children with neuroblastoma (NBL) [1,2]. BM infiltration at diagnosis and persistence during treatment is predictive of poor outcome [3,4,5,6,7]. In the current protocols, BM infiltration at diagnosis and during treatment is assessed by histology or (immuno)cytology, and more sensitive detection of tumor cells by reverse-transcriptase quantitative polymerase chain reaction (RT-qPCR) is under investigation [8,9,10]. Multiple studies showed that detection of MRD with various mRNA markers throughout and post-induction therapy could be prognostic of outcome [3,4,11,12,13].

We have previously described paired-like homeobox 2b (*PHOX2B*) as a sensitive and NBL specific mRNA marker for minimal residual disease (MRD) detection by RT-qPCR, with high expression in NBL tumors and no expression in normal BM and peripheral blood (PB) [8]. However, the marker shows variable expression levels between tumors and not all tumors can be identified with only *PHOX2B.* Therefore, we then demonstrated that the addition of other markers contributes to more sensitive MRD detection [9]. Based on high expression in NBL tumors and low/no expression in normal BM or PB, we optimized two mRNA marker panels, one specific for BM, the other specific for PB. *PHOX2B*, tyrosine hydroxylase (*TH*), cholinergic receptor nicotinic alpha 3 (*CHRNA3*), and growth associated protein 43 (*GAP43*) are part of the BM panel. *PHOX2B*, *TH*, *CHRNA3* and dopamine beta hydroxylase (*DBH*) form the PB panel [8,9]. 

Several patients scoring negative for MRD detection with the above-mentioned adrenergic (ADRN) marker panels still experience relapse [4,13]. Epithelial to mesenchymal transition (EMT) is a process that plays a role in tumorigenic progression, resulting in an increased motility of cancer cells. EMT induces the conversion of cells from an epithelial to mesenchymal (MES) phenotype, and assists invasion, dissemination and thereby disease progression [14]. Metastasis and therapy resistance are results of EMT in diverse cancers. NBL tumors can contain both ADRN and MES cells, an enrichment of MES cells occurs in post-treatment and recurring tumors. The MES cells lack expression of regularly used NBL MRD markers, *PHOX2B* and *DBH* [15]. Since MES NBL cells might not be detected with the present MRD panel, a MES marker panel for MRD detection was developed. Periostin (*POSTN*) and paired related homeobox 1 (*PRRX1*) are MES specific. Together with flavin containing monooxygenase 3 (*FMO3*), which is not expressed in NBL cells but is expressed in mesenchymal stromal cells and used to differentiate these from MES, these markers form the MES marker panel. High expression of *POSTN* and *PRRX1* and low/no expression of *FMO3* can identify MES NBL cells in BM and PB [13].

Altogether these studies show that multiple targets are required for reliable MRD detection. However, patient-derived samples are precious since NBL patients are young of age, and thus only small volumes of BM or PB can be sampled. Multiplex (MPX) RT-qPCR reduces the sample volume required for detection of markers, and saves costs and time, which is important for clinical implementation. In this study, we aim to generate a workflow for a marker panel of NBL specific mRNA targets by MPX RT-qPCR for MRD detection implementation in clinical care of neuroblastoma.

## 2. Results

### 2.1. Optimization of RT-qPCR Setting

In order to ascertain compatibility in a MPX setting, the primers and probes for the mRNA markers were redesigned (sequences are listed in Table S1). The fluorophores FAM, Yakima Yellow™(YY) and Dragonfly Orange™ (DFO) were chosen to match the different channels available in the Viia7, with the appropriate Black Hole Quenchers®(BHQ). qPCR Master Mix containing ROX as a reference control was replaced by a master mix with Mustang Purple as the reference control, to eliminate the efflux of the Dragonfly orange fluorophore into the ROX filter channel. We observed a disturbance of RT-qPCR amplification plots through fluorescence quenching, which was caused by interaction between Mustang Purple and dithiothreitol (DTT) [16] from the reverse transcription mix (containing first strand buffer, DTT, dNTPs, random primers, RNAseout and MMLV). We therefore replaced this reverse transcriptase mix with the High-Capacity RNA-to-cDNA™ Kit (Applied Biosystems, Foster City, CA, USA).

### 2.2. Expression of Singleplex vs. MPX on Neuroblastoma Cell Lines

**Figure 1:**
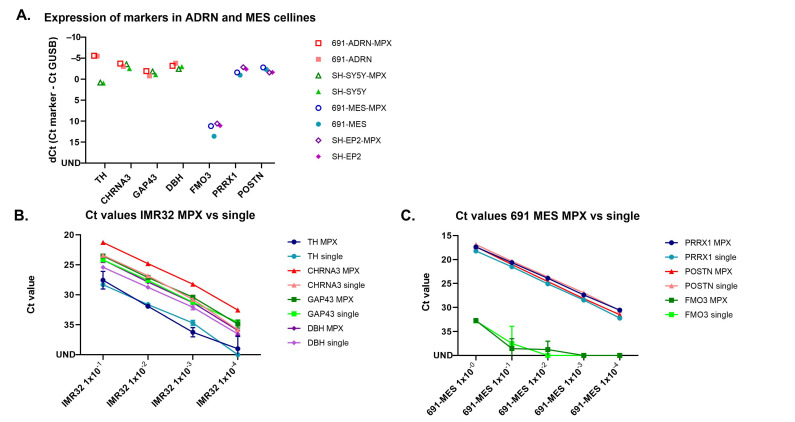
Expression of ADRN and MES panel markers in ADRN and MES cell lines with singleplex and multiplex methods (**A**) ADRN cell lines 691-ADRN and SH-SY5Y in red and green, respectively. MES cell lines 691-MES and SH-EP2 in blue and purple, respectively. Expression with multiplex method in open symbols, expression with singleplex method in filled symbols. UND = undetermined; (**B**) Dilution curve of IMR32, ADRN markers tested in MPX and singleplex; (**C**) Dilution curve of 691-MES, MES markers tested in MPX and singleplex.

The expression of the ADRN and MES marker panel was measured in two isogenic neuroblastoma cell line pairs (691-MES/691-ADRN and SH-EP2/SH-SY5Y) with both singleplex and MPX RT-qPCR methods (Figure 1A). The expression levels of the ADRN panel on the ADRN cell lines and of the MES panel on MES cell lines were comparable in singleplex and MPX setting. The dilution curves of IMR32 (ADRN cell line) and 691-MES (MES cell line) display comparable sensitivity of the MPX RT-PCR and singleplex method (Figure 1B,C).

### 2.3. TH in MPX RT-qPCR Occasionally Showed Amplification of gDNA

When we proceeded to testing of the MPX panels in control and patient samples, we observed an aspecific amplification curve for *TH* in a fair number of samples, often resulting in a low Ct value. Sanger sequencing of the product revealed the genomic TH sequence. Furthermore, with this assay we detected amplifications on unconverted RNA as well (Figure S1), suggesting the detection of gDNA instead of cDNA. This was independent of RNA isolation method and DNAse treatment (included in PAXgene RNA isolation) (Figure S2). Of note, these aspecific curves for TH were also observed in singleplex RT-qPCR (Figure S3). Since these primer and probe sequences for *TH* are widely used within Europe [17,18], we were hesitant to redesign this assay. With a visual inspection of the amplification and multicomponent plot, as well as with the use of the AMPSCORE (indicating if the amplification is in the linear region), the CQCONF score (indicating the calculated confidence for the Ct value of the well) and dRN results, all provided within the QuantStudio software [19], we were able to distinguish ‘true’ from ‘false’ amplifications. Examples of the different amplification plots and multicomponent plots in MPX and singleplex RT-qPCR, as well as qPCR on the samples without reverse transcriptase (RT) can be found in Figure S3. The Ct values, AMPSCORE, CQCONF score and ΔRn values of these examples are listed in Table S2. The proposed required conditions for the confirmation of true TH amplifications can be found in Figure S4.

### 2.4. Setting a Threshold for Positivity for ADRN and MES mRNA MPX in BM and PB

**Figure 2:**
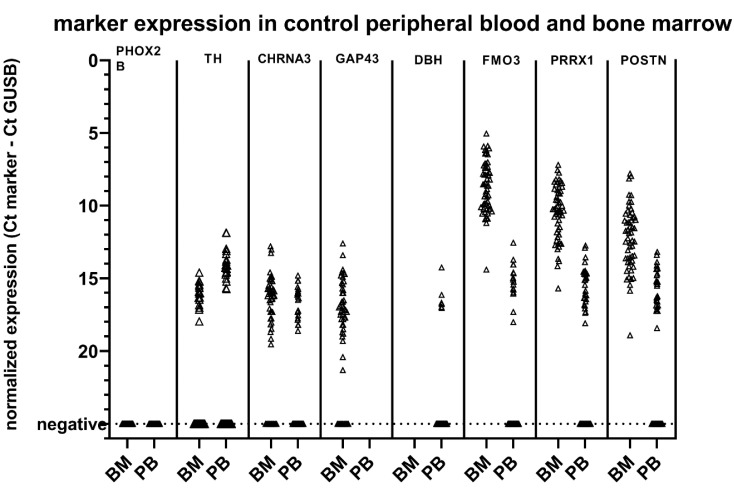
Marker expression with the multiplex method in control peripheral blood (PB) and bone marrow (BM).

To establish new thresholds for positivity for the MPX panel, we determined expression MPX panels in control BM (*n* = 54) and PB (*n* = 50) (Figure 2). It should be noted that the Ct of *GUSB* was on average 2.8 and 2.0 Ct lower compared to data obtained with control samples in our previous study for BM and PB, respectively (Table S3). In the current experiments, we were able to include samples with concentrations as low as 80 ng of total RNA for cDNA synthesis, which still resulted in a *GUSB* Ct value of 24 (4.5 ng of RNA was used for *GUSB* RT-qPCR, in duplicate). This seemed to be a result of the more efficient cDNA synthesis method using the High-Capacity RNA-to-cDNA™ Kit we used in the present study, as the RNA isolation method was similar to before. Applying the rules adapted from the European Study Group on MRD detection in ALL [20], we defined a threshold for positivity for the ADRN (BM and PB) and MES (PB only) markers as ΔCt_sample_ < 3.0 Ct than median ΔCt_control tissue_. Patient samples were scored positive when Ct_sample_ < 40 and mean ΔCt_sample_ < 3.0 Ct than median ΔCt_control tissue_. As described previously [8,9], *PHOX2B* was not expressed in any of the control samples. Based on the levels of expression in control BM and PB, a threshold was set for positivity for the other ADRN markers, and for the MES markers in PB (Table 1). For the MES markers in BM, *FMO3* positivity was taken into account resulting in the thresholds in Table 1. The number of control samples with expression of our markers was slightly increased, possibly due to the more efficient cDNA synthesis. However, ΔCt results were comparable to earlier established thresholds (Table S3), with the exception of *FMO3* in BM, which showed an expression of 2.2 Ct lower than previously published data [9,13]. Of note, *FMO3* amplifications were not detected in unconverted RNA, excluding the possibility that detection of gDNA resulted in lower Ct values. Based on the expression in control samples, we defined the threshold for PRRX1 in BM as Ct*_PRRX1_* − Ct*_GUSB_* < 9.0 and Ct*_PRRX1_* − Ct*_FMO_*_3_ < −1, and for POSTN in BM as Ct*_POSTN_* – Ct*_GUSB_* < 9.0 and Ct*_POSTN_* − Ct*_FMO_*_3_ < 1. With these thresholds, none of the controls were positive for the MES markers. 

### 2.5. Comparison of Singleplex vs. MPX RT-qPCR in Patient Samples

**Figure 3:**
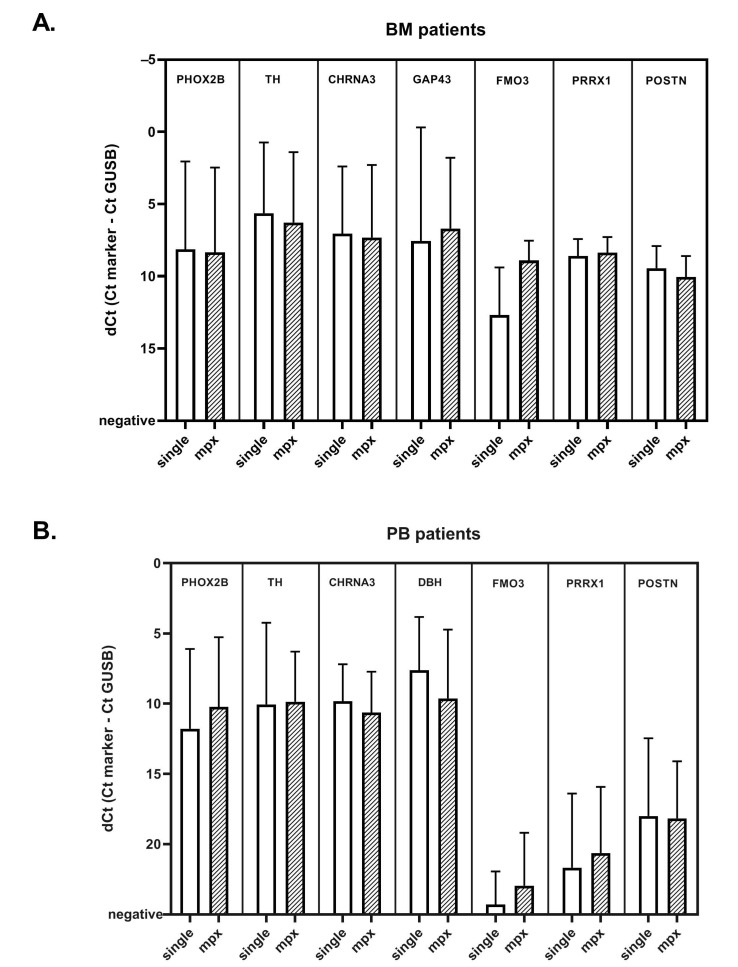
Mean normalized Ct values in BM (**A**) and PB (**B**) of patients with neuroblastoma, for singleplex and multiplex methods. Bar graph showing the mean normalized Ct value (ΔCt = Ct_marker_ – Ct*_GUSB_*) for ADRN and MES markers in singleplex and multiplex methods, in A: bone marrow (BM) and B: peripheral blood (PB) samples of patients with neuroblastoma. Whiskers indicate the standard deviation.

To compare the expression between singleplex and MPX RT-qPCR assays, we performed MPX RT-qPCR on 21 PB and 24 BM samples from patients with NBL (Table S4), which were previously measured in single RT-qPCR settings for ADRN and MES mRNA markers. Overall, ΔCts for all markers were comparable in BM and PB, with the exception of *FMO3* in BM samples (Figure 3). Individual correlation plots for each sample per marker can be found in Figure S5. The ΔCt of ADRN markers showed a good correlation between singleplex and MPX in both BM and PB. The MES markers were infrequently detected in PB. *POSTN* and *PRRX1* show a good correlation in BM, but the ΔCt for *FMO3* was consistently lower in BM. We therefore tested for *FMO3* in 100 additional BM samples from patients with NBL in parallel for both singleplex and MPX. While the median Ct was 3.2 lower in the MPX assay, the Ct values in singleplex and MPX do seem to correlate (Figure S6). Subsequently, in this cohort of 21 PB and 24 BM samples of NBL patients, we evaluated the performance of the MPX compared to singleplex RT-qPCR in considering samples positive. The results obtained in this analysis are summarized in Table 2, more detailed 2x2 tables per marker can be found in Table S5. All BM samples were positive for *PHOX2B,* and *TH* identified the same samples in MPX and singleplex. *GAP43* was positive in 6 samples in MPX, and negative in singleplex, and one sample was positive for *CHRNA3* in singleplex and negative in MPX, and vice versa, but since all samples were positive for *PHOX2B*, this did not have any effect on positivity for the panel as a whole. The MES marker *POSTN* scored positive slightly more often in the MPX setting (7 samples, compared to 6 in singleplex). Next, we focused on PB samples. Out of the two samples that were *PHOX2B* negative in singleplex, only one was *PHOX2B* positive in MPX. Since this sample scored positive for *DBH* in singleplex (ΔCt of 12.1; threshold 15.0), this was already considered positive for the ADRN panel. As the primer and probe sequences of *PHOX2B* are not altered, and *PHOX2B* is still tested as a single marker, this can be a result of the more efficient cDNA synthesis method. The only sample that now tested negative for *PHOX2B* was only ADRN positive in singleplex because of a very low DBH expression, on the borderline of positivity (ΔCt of 14.7; threshold 15.0). Despite the comparable ΔCt values in singleplex and MPX, *TH* was less frequently positive in MPX, due to the lowered threshold. None of these *TH*-discrepant samples had a negative MPX result when the whole ADRN panel was taken into account, as *PHOX2B* was positive in all these samples. None of the PB samples were considered positive for the MES panel. Overall, these results indicate that the MPX RT-qPCR reliably detects presence of NBL mRNA in this cohort of BM and PB patient samples.

## 3. Discussion

mRNA detection by RT-qPCR is a very sensitive and promising technique for detection of disseminated neuroblastoma cells at diagnosis, and MRD during therapy. We previously showed that our panel of mRNA markers is superior to one single marker in the detection of neuroblastoma cells and therefore can transcend heterogeneity of the neuroblastoma tumor cells [9,21]. With the addition of our mesenchymal mRNA markers, we are able to detect neuroblastoma cells undergoing EMT during therapy [13,15,22] and can thus prevent false negativity. These EMT markers can be further used to study EMT in other tumor entities. However, often the sample quantity required to test multiple mRNA markers is limited. As the field of PCR-based mRNA detection is evolving rapidly, with different PCR approaches, RNA isolation methods, and new mRNA markers, we looked to optimize our workflow to increase the yield of the samples and we aimed to combine various mRNA markers in less RT-qPCR reactions. In this study, we redesigned the primer and probe sequences and optimized cDNA synthesis and RT-qPCR conditions. We tested control PB samples from healthy donors and BM samples from children with ALL in complete molecular remission to test for illegitimate expression of the markers. With the exception of *FMO3*, all redesigned markers in MPX showed a similar expression level compared to the old designs in singleplex in control samples [9]. The lower Ct value of the MPX RT-qPCR for FMO3 can be explained by the fact that the MPX *FMO3* assay is designed in exon 5 and 6, while the singleplex *FMO3* was designed in exon 2 and 3 [13]. Exon 3 is not part of the transcript NM_001319174.2 *Homo sapiens* flavin containing dimethylaniline monoxygenase 3 (*FMO3*), transcript variant 4, mRNA [23,24], and the RT-qPCR assay designed in exon 3 can thus result in a lower expression detected. Furthermore, the current amplicon for FMO3 in MPX RT-qPCR is shorter (86 bp) compared to the previous amplicon (113 bp), and the previous singleplex primers scored high for self-complementarity, which may explain why the MPX RT-qPCR is more efficient and results in lower Ct values [25]. For all markers tested in MPX, we defined new thresholds for positivity based on the expression of the markers in the control samples. There are multiple approaches to score patient-derived samples by RT-qPCR. Specifically, expression in control samples can be used to establish a threshold [8,9], or alternatively a cut-off for low and high expression can be established based on outcome of a patient cohort [3]. The advantage of the use of thresholds for positivity, based on control samples, is that these are not susceptible to treatment related changes and moreover results in a higher specificity of the RT-qPCR. With these new defined thresholds, we analyzed 21 PB and 24 BM samples from patients with neuroblastoma. MPX RT-qPCR resulted in a positive result for the ADRN marker panel in all 24 BM that were positive for the singleplex RT-qPCR, and identified one additional MES positive BM sample. Apart from one sample that was considered borderline positive in singleplex, based on a ΔCt of 14.7 for *DBH* (threshold 15.0), our findings confirm the comparability of the MPX and singleplex RT-qPCR. We are presently confirming the sensitivity and prognostic relevance of these markers with multiplex RT-qPCR in an ongoing prospective validation study.

One unexpected technical finding during the optimization of the MPX RT-qPCR was the overestimation of target expression levels, due to fluorescence quenching of the passive reference dye Mustang Purple by DTT [16]. We therefore replaced the reverse transcriptase method to one without DTT. The improved cDNA synthesis with the High-Capacity RNA-to-cDNA™ kit overall resulted in a decrease of 2.3 Ct of *GUSB*. Since we exclude samples that have a *GUSB* Ct value > 25 (corresponding to less than 5000 copies) [8,26,27], this facilitates the possibility to also test samples with a low RNA concentration. As the RNA yield in BM and PB samples collected during therapy is often low, this results in many excluded samples in larger studies [3], even when optimized standard operating procedures are in place. With the two newly developed MPX only half of the cDNA amount compared to the singleplex approach is required. Furthermore, combined with the improved cDNA synthesis, we are able to test 8 different mRNA markers, in all samples, even in those samples of low RNA quantity. 

As we developed and optimized the MPX panels, we observed an aspecific amplification curve for *TH* in a fair number of samples, often resulting in a low Ct value. When we analyzed the same samples in singleplex, we still observed this aspecific amplification. We confirmed that *TH* gDNA was present in these samples after RNA isolation, even with DNAse treatment, and is causing this aspecific amplification. While we are able to distinguish between true and false positive *TH* amplifications with the use of QuantStudio software, these findings stress the need to carefully analyze amplifications when developing and optimizing RT-qPCR assays, to avoid false-positive samples.

## 4. Materials and Methods 

### 4.1. Patients and Samples

We analyzed stored remains from BM and PB samples at diagnosis and during treatment from NBL patients with high-risk neuroblastoma, treated in accordance with the German NB2004 or Dutch NBL2009 trial [28,29]. The study was approved by the medical ethics committees (Academic Medical Center, Amsterdam, the Netherlands; MEC07/219#08.17.0836) and the University of Cologne. Samples were processed within 24 h after collection and stored in PAX tubes at −20 °C (Qiagen, Venlo, the Netherlands), or in Trizol (Invitrogen, Carlsbad, CA, USA) or RNAbee (Campro Scientific, Berlin, Germany) at −80 ℃. To establish thresholds for the newly designed markers, stored remains from 54 BM samples from patients with childhood acute lymphoid leukemia in molecular remission and 50 PB samples from healthy volunteers were used. Neuroblastoma cell lines SH-EP2, SH-Sy5Y, IMR32, 691-MES and 691-ADRN cells were cultured as previously described [30,31].

### 4.2. RNA Isolation and cDNA Synthesis

Isolation of mRNA from samples was done through the PAXgene Blood Kit (QIAGEN, Venlo, The Netherlands), through the RNAbee method (Campro Scientific, Berlin, Germany) or with Trizol (Invitrogen, Carlsbad, CA, USA), according to the manufacturer’s instructions. Concentration and quality of RNA were measured by an ND-1000 spectrophotometer (ThermoFisher Scientific, Waltham, MA, USA). For the samples tested in singleplex, cDNA synthesis was performed as described previously [32]. For the samples tested in MPX, synthesis of cDNA was done by the High-Capacity RNA-to-cDNA™ Kit (Applied Biosystems, Foster City, CA, USA), using 2–3 µg of RNA, 15 µL of RT Buffer Mix and 1.5 µL Enzyme Mix, in a total reaction volume of 30 μL and incubated at 37 °C for 60 min. Finally, the reverse transcriptase was inactivated by heating to 95 °C for 5 min, and the volume was diluted with H_2_O to 75 µL. For samples with an RNA concentration < 20 ng/μL, we used up to 40 μL of RNA together with 44.5 µL of RT Buffer Mix and 4.5 µL Enzyme Mix and did not dilute with H_2_O. cDNA samples were used immediately or stored at −20 ℃. 

### 4.3. Multiplex Real-Time qPCR and Sequencing

Primers and probe for reference gene glucuronidase beta (*GUSB*) and *PHOX2B* have been published previously and continued to be used as single RT-qPCR markers [8,26]. *TH*, *GAP43* and *CHRNA3* were combined as the MPX BM panel, *TH*, *CHRNA3* and *DBH* were combined as the MPX PB panel. *POSTN*, *PRRX1* and *FMO3* were combined as the MPX MES panel. *TH* primers and probe sequences have been described before [33]. For all other markers, new primers and probes were designed for compatibility in a MPX setting. Primers and probes were designed with Primer Express software (version 1.5; ThermoFisher Scientific, Waltham, MA, USA) and Oligo7 (Molecular Biology Insights, Colorado Springs, CO, USA) and synthesized by Eurogentec (Liege, Belgium) and are listed in Supplemental Table S1. RT-qPCR was performed on the Viia7 (Applied Biosystems, Foster, CA, USA), and analysis was performed using QuantStudio software version 1.4 (Applied Biosystems, Foster, CA, USA). The probe quencher and fluorescent reporter were chosen for the channels available in Viia7. Reactions were carried out in 20 µL (10 µL TaqMan^TM^ Multiplex Master Mix (Applied Biosystems, Foster, CA, USA), 300 nM forward and reverse primer and 200 nM probe and 5 µL cDNA). In all RT-qPCR reactions, initial heating was done for 20 s at 95 °C, followed by 50 cycles of 1 s at 95 °C and 20 s at 60 °C. All RT-qPCR reactions were performed in triplicate (except *GUSB*, which was performed in duplicate), and mean values were used for analysis. A given sample scored negative if the Ct value was 40 or greater, with the exception of PHOX2B (Ct ≤ 50). Sequencing of PCR products was performed as previously described [32]. 

### 4.4. Data Analysis

For both singleplex and MPX, expression was normalized to *GUSB* expression using the following equation: [normalized threshold cycle (ΔCt) = Ct_marker_ – Ct_GUSB_]. For the singleplex RT-qPCR, positivity of samples was scored according to earlier published thresholds [9,13]. For the MPX RT-qPCR, thresholds for positivity in control BM and PB were determined on the basis of the expression in the control sample (see Results). While we continue to test *PHOX2B* as a single marker, we changed the cDNA synthesis method and the RT-qPCR Master Mix, and therefore determined the expression of *PHOX2B* in control- and patient samples. A threshold for positivity for the ADRN markers was defined as ΔCt_sample_ < 3.0 Ct than median ΔCt_control tissue_. For the MES markers, the expression of *FMO3* was taken into account [13].

## 5. Conclusions

In conclusion, we developed a reliable and sensitive MPX RT-qPCR for a panel of ADRN and MES neuroblastoma MRD markers. NBL patients are young, and the volume of BM and PB that can be sampled is limited. As neuroblastoma tumors can be phenotypically heterogeneous, a panel of mRNA markers improves the sensitivity of MRD monitoring using RT-qPCR. By testing these markers in MPX, we save time, resources and make optimal use of these precious samples. 

## Figures and Tables

**Table 1:** Median normalized expression levels of ADRN and MES panel by multiplex RT-qPCR in control BM (*n* = 54) and control PB (*n* = 50).

Marker	BM			PB		
	Positive BM Samples	Expression (Median + IQR)	Threshold	Positive PB Samples	Expression (Median + IQR)	Threshold
*PHOX2B*	0/54			0/50		
*TH*	17/54	16.0 (0.9)	13.0	21/50	14.3 (0.9)	11.3
*CHRNA3*	42/54	16.0 (1.7)	13.0	21/50	16.4 (1.7)	13.4
*GAP43*	42/54	17.0 (2.3)	14.0			
*DBH*				6/50	16.7 (0.7)	13.7
*FMO3*	54/54	8.6 (2.7)		16/50	15.2 (1.4)	
*PRRX1*	54/54	10.3 (2.8)	9.0 *	30/50	15.3 (1.7)	12.3 **
*POSTN*	54/54	12.1 (3.0)	9.0 *	27/50	15.4 (2.0)	12.5 **

All samples represent the median (±interquartile range (IQR)) of normalized Ct values (ΔCt = Ct_marker_ – Ct*_GUSB_*). * Threshold for *PRRX1* in BM is defined as ΔCt < 9.0 and Ct*_PRRX1_* − Ct*_FMO_*_3_ < −1. Threshold for *POSTN* in BM is defined as ΔCt < 9.0 and Ct*_POSTN_* − Ct*_FMO_*_3_ < 1. ** Threshold for *PRRX1* and *POSTN* in PB is defined as ΔCt < 12.3 and 12.5 respectively, and no expression of *FMO3*. BM = bone marrow, PB = peripheral blood.

**Table 2:** Comparison of singleplex and multiplex testing of patient bone marrow (BM) and peripheral blood (PB) samples.

Marker	BM (*n* = 24)		PB (*n* = 21)	
	MPX Positive BM Samples	Singpleplex Positive BM Samples	MPX Positive PB Samples	Singleplex Positive PB Samples
*PHOX2B*	24	24	20	19
*TH*	22	22	13	18
*CHRNA3*	20	20	16	16
*GAP43*	24	18		
*DBH*			18	21
Total ADRN	24 *	24	20 **	21
*PRRX1*	9	9	0	0
*POSTN*	7	6	0	0
Total MES	11 ***	10	0	0

* Sensitivity of 100% compared to the singleplex RT-qPCR in the BM cohort. ** Sensitivity of 95% compared to singleplex RT-qPCR in the PB cohort. *** Sensitivity of 80% compared to singleplex RT-qPCR in the BM cohort.

## Data Availability

The authors confirm that the data analyzed during this study are available from the corresponding author, G.A.M.T., upon reasonable request.

## Supplementary Materials

The following are available online at https://www.mdpi.com/2072-6694/13/1/150/s1, Figure S1: Example of an amplification curve with low ΔRN, low Ct value, cDNA and RNA without RT, Figure S2: Detection of gDNA in RNA of IMR-32 without RT, Trizol isolation method compared to PAXgene isolation method (including DNAse treatment), Figure S3: Examples of different amplification curves for *TH* in MPX RT-qPCR, qPCR (without RT) and singleplex RT-Qpcr, Figure S4. Required conditions for the evaluation of TH- amplification curves in MPX RT-qPCR, Figure S5. Correlation plots for individual samples per marker, Figure S6. Correlation plot for *FMO3* in MPX and singleplex in 100 BM samples, Table S1: Primers and probe sequences for multiplex method, Table S2. Examples Ct, ΔCt, AMPSCORE, CQCONF score and ΔRn results of MPX RT-qPCR, RNA qPCR and singleplex RT-qPCR, Table S3. Comparison of ΔCt and Ct results between multiplex and singleplex in control tissue, Table S4. Sample and patient characteristics of the cohort used to compare singleplex and multiplex RT-qPCR, Table S5. Detailed results of the comparison between MPX and singleplex RT-qPCR in a cohort of 24 bone marrow and 21 blood samples.

## Abbreviations

ADRN	adrenergic (neuroblastoma)
BHQ	Black Hole Quencher
Ct	cycle threshold
DFO	Dragonfly Orange™
dNTP	deoxyribonucleotide triphosphate
DTT	dithiothreitol
EMT	epithelial to mesenchymal transition
MES	mesenchymal (neuroblastoma)
MMLV	Moloney Murine Leukemia Virus
MPX	multiplex RT-qPCR
NBL	neuroblastoma
PB	peripheral blood
YY	Yakima Yellow™

## Genes

*CHRNA3*	cholinergic receptor nicotinic alpha 3
*DBH*	dopamine beta hydroxylase
*FMO3*	flavin containing monooxygenase 3
*GAP43*	growth associated protein 43
*GUSB*	glucuronidase beta
*PHOX2B*	paired-like homeobox 2b
*POSTN*	periostin
*PRRX1*	paired related homeobox 1
*TH*	tyrosine hydroxylase
