# Supplementary material for: Specific and Sensitive Detection of Neuroblastoma mRNA Markers by Multiplex RT-qPCR
Source: Cancers (Basel). 2021 Jan 5;13(1):150. doi: 10.3390/cancers13010150 (PMC7796198; doi:10.3390/cancers13010150)
Supplement: Supplementary file 1 [file cancers-13-00150-s001.pdf]

Supplementary Materials: Specific and Sensitive Detection of Neuroblastoma mRNA Markers by Multiplex RT-qPCR

Lieke M.J. van Zogchel, L. Zappeij-Kannegieter, Ahmad Javadi, Marjolein Lugtigheid, Nina U. Gelineau, Nathalie S.M. Lak, Danny A. Zwijnenburg, Jan Koster, Janine Stutterheim, C. Ellen van der Schoot and Godelieve A.M. Tytgat

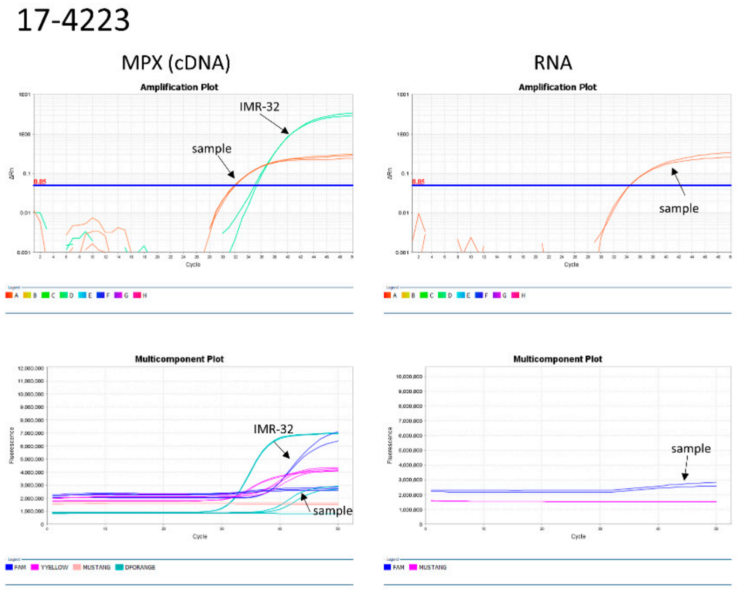

Figure S1. Example of an amplification curve with low ΔRN, low Ct value, cDNA and RNA without RT. Amplification plots (**upper row**) and multicomponent plots (**lower row**) are shown for cDNA in MPX RT-qPCR (**left column**) and RNA without RT in qPCR (**right column**). In the multicomponent plots, TH amplifications are shown by the blue line (FAM-channel). Sample and lowest dilution of IMR-32 are indicated by label and arrow. An amplification curve of sample 17-4223 is visible in MPX RT-qPCR, resulting in a low Ct value. However, in the multicomponent plot a very minimal increase in fluorescence is seen. When qPCR without RT is performed on this sample, the same amplification curve and multicomponent plot is shown, suggesting the detection of gDNA in MPX RT-qPCR.

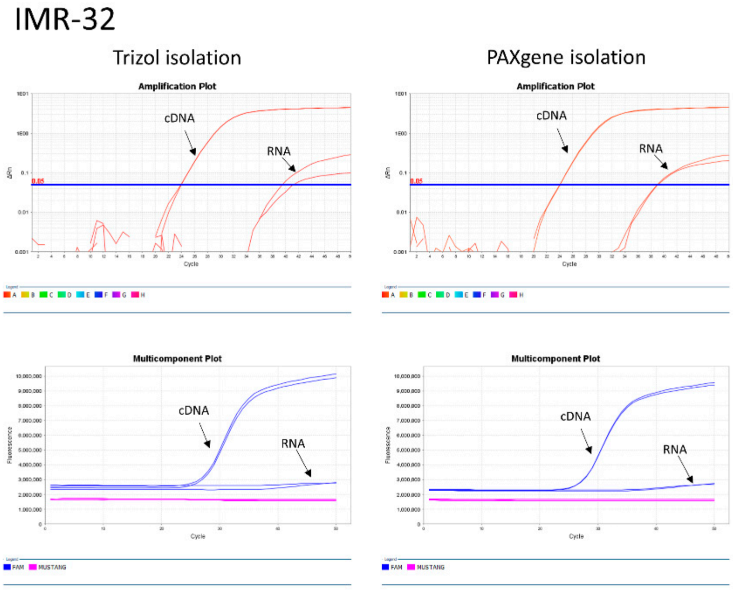

Figure S2. Detection of gDNA in RNA of IMR-32 without RT, Trizol isolation method compared to PAXgene isolation method (including DNAse treatment). Amplification plots (**upper row**) and multicomponent plots (**lower row**) are shown for cDNA in MPX RT-qPCR (**left column**) and RNA without RT in qPCR (**right column**). In the multicomponent plots, TH amplifications are shown by the blue line (FAM-channel). cDNA (with RT) and RNA (without RT) of IMR-32 are indicated by label and arrow. An amplification curve of IMR-32 RNA without RT, isolated either by Trizol method or PAXgene is visible in MPX RT-qPCR. However, in the multicomponent plot, a very minimal increase in fluorescence is seen, suggesting the detection of gDNA.

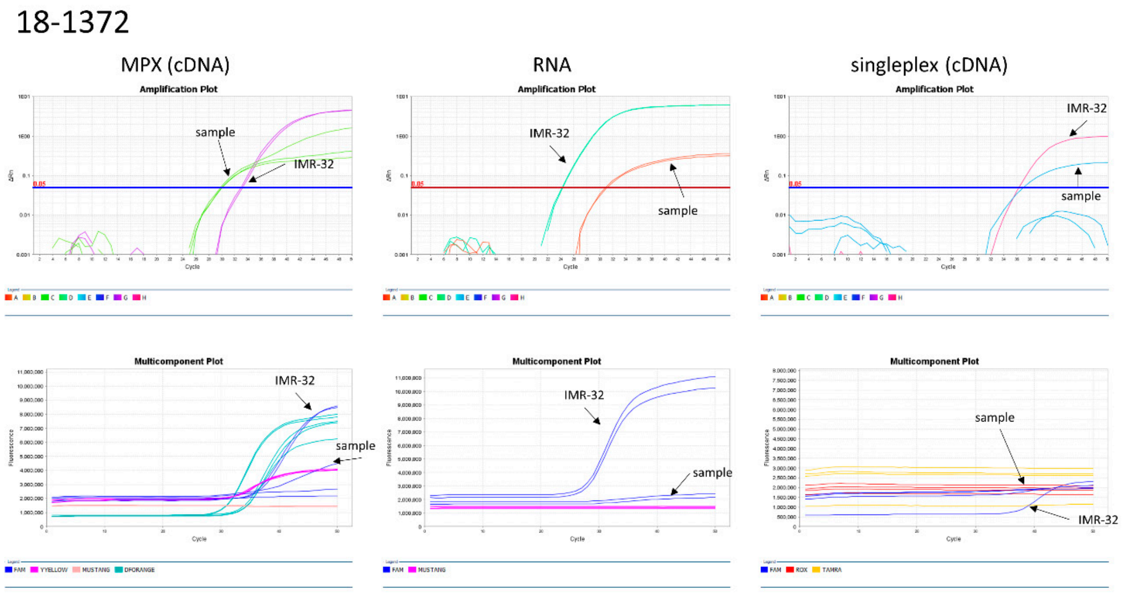
18-1372
MPX (cDNA)
RNA
singleplex (cDNA)
Amplification Plot
sample
IMR-32
Amplification Plot
IMR-32
sample
Amplification Plot
IMR-32
sample
Multicomponent Plot
IMR-32
sample
Multicomponent Plot
IMR-32
sample
Multicomponent Plot
sample
IMR-32

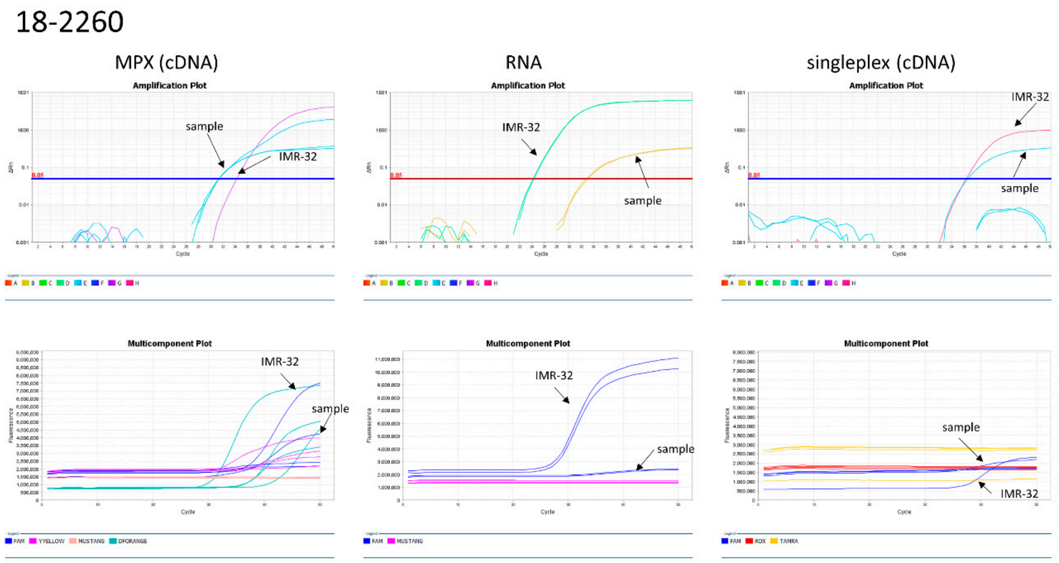
18-2260
MPX (cDNA)
RNA
singleplex (cDNA)
Amplification Plot
sample
IMR-32
Amplification Plot
IMR-32
sample
Amplification Plot
IMR-32
sample
Multicomponent Plot
IMR-32
sample
Multicomponent Plot
IMR-32
sample
Multicomponent Plot
sample
IMR-32

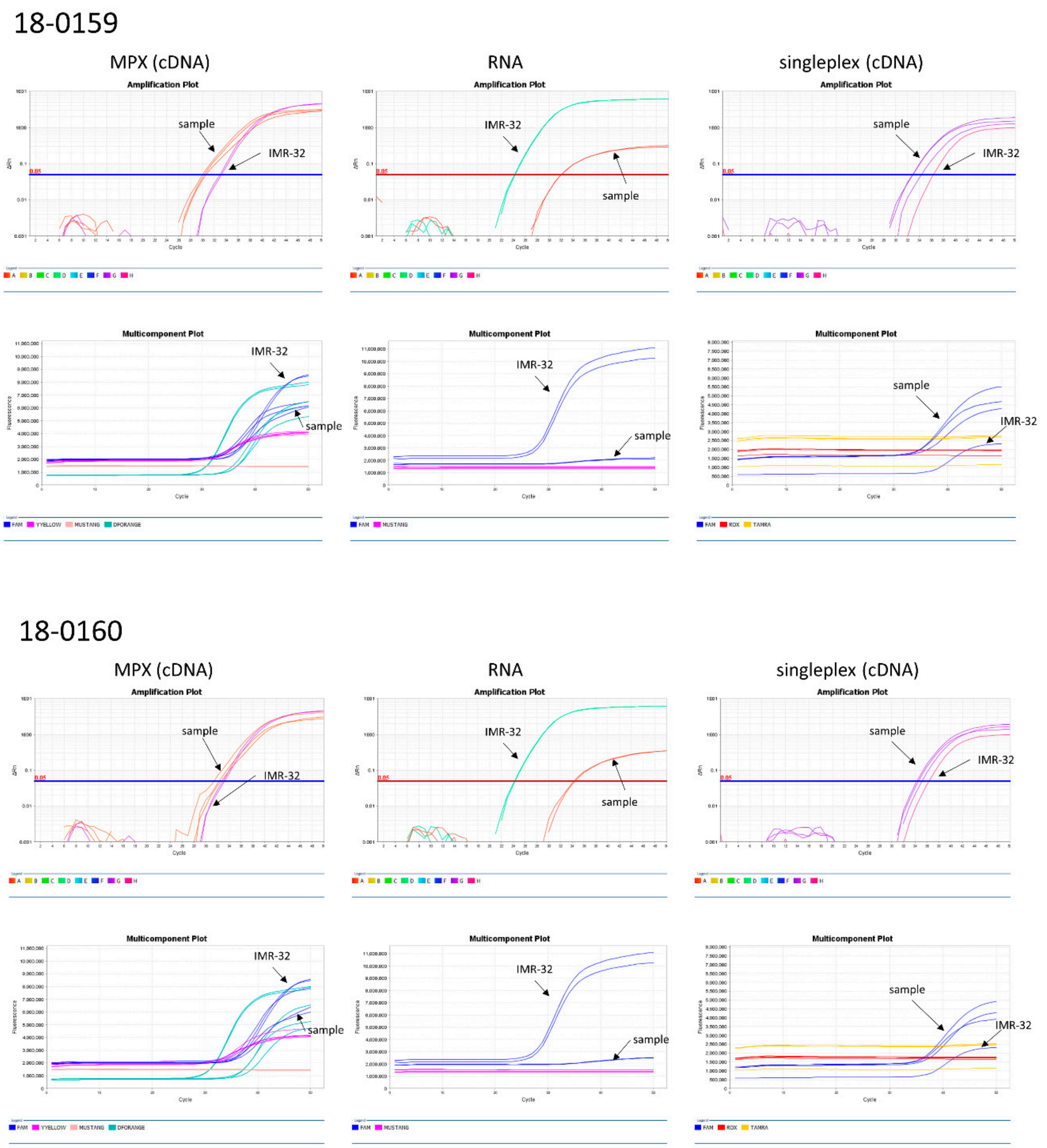

Figure S3. Examples of different amplification curves for TH in MPX RT-qPCR, qPCR (without RT) and singleplex RT-qPCR. Amplification plots (**upper row**) and multicomponent plots (**lower row**) are shown for cDNA in MPX RT-qPCR (**left column**), RNA without RT in qPCR (**middle column**) and cDNA in singleplex RT-qPCR (**right column**). cDNA for singleplex RT-qPCR was synthesized as previously described [1]. In the multicomponent plots, TH amplifications are shown by the blue line (FAM-channel). Sample and lowest dilution of IMR-32 are indicated by label and arrow. For samples 18-1372 and 18-2260 amplification curves are seen, with various ΔRn results. The amplification curves of samples 18-0159 and 18-0160 result in higher ΔRn values (>1). For all samples, amplification is observed in RNA samples without RT. Singpleplex RT-qPCR shows for 18-1372 and 18-2260 amplifications in single, but with ΔRn < 1. In singpleplex RT-qPCR 18-0159 and 18-0160 show amplifications in triplicate, with ΔRn < 1.

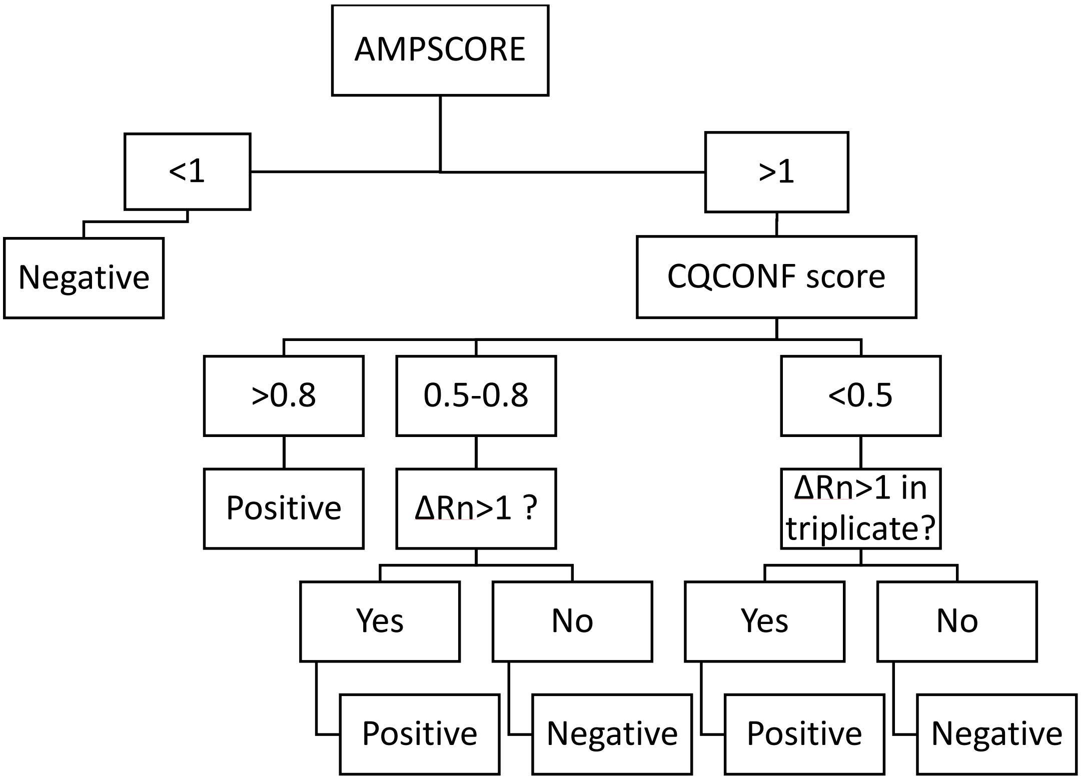

Figure S4. Required conditions for the evaluation of TH- amplification curves in MPX RT-qPCR. In a set of 16 ambiguous samples, all 8 'true positives' had a ΔRn >1 in triplicate, ΔAMPSCORE (AMPSCORE cDNA – AMPSCORE RNA) > 0.2 and the ΔΔRn (ΔRn cDNA – ΔRn RNA) > 1, which all the 'false positives' had not. Based on these results, together with the recommended AMPSCORE and CQCONF score, we propose these conditions for the evaluation of TH-amplification curves in MPX RT-qPCR.

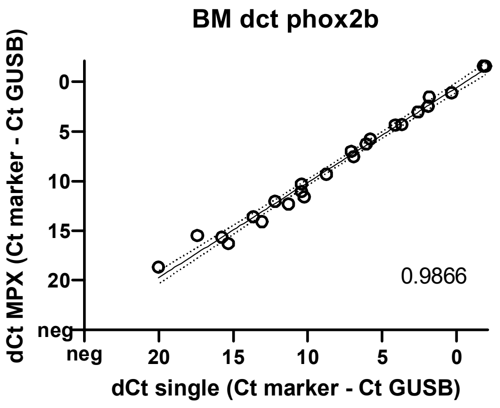
BM dct phox2b
dCt MPX (Ct marker - Ct GUSB)
dCt single (Ct marker - Ct GUSB)
0.9866

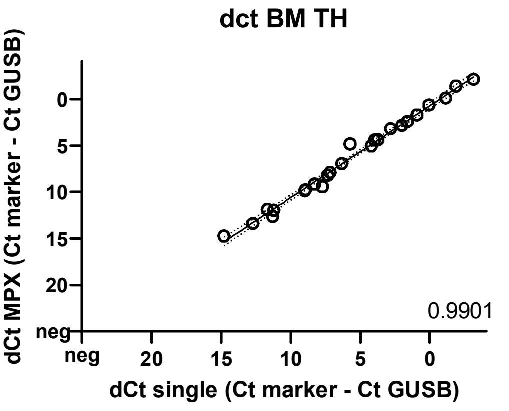
dct BM TH
dCt MPX (Ct marker - Ct GUSB)
dCt single (Ct marker - Ct GUSB)
0.9901

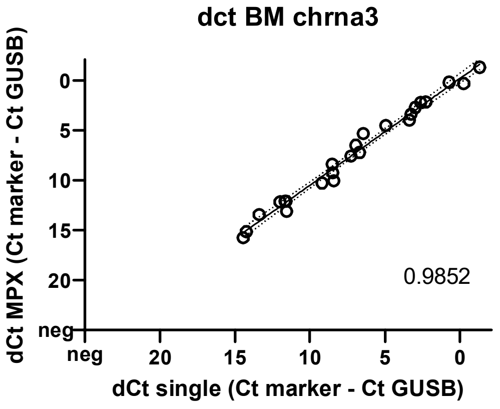
dct BM chrna3
dCt MPX (Ct marker - Ct GUSB)
dCt single (Ct marker - Ct GUSB)
0.9852

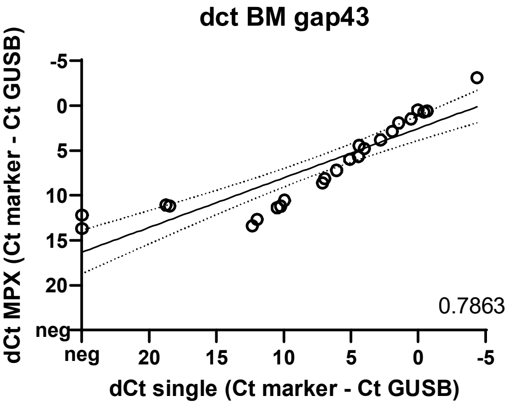
dct BM gap43
dCt MPX (Ct marker - Ct GUSB)
dCt single (Ct marker - Ct GUSB)
0.7863

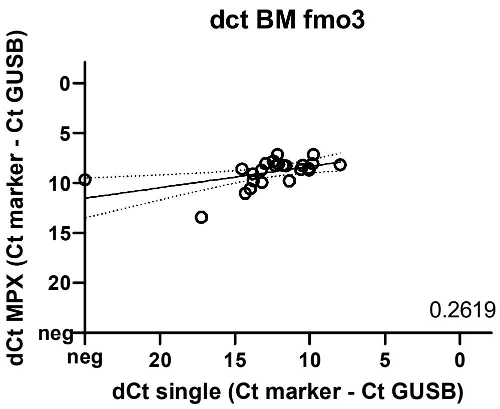
dct BM fmo3
dCt MPX (Ct marker - Ct GUSB)
dCt single (Ct marker - Ct GUSB)
0.2619

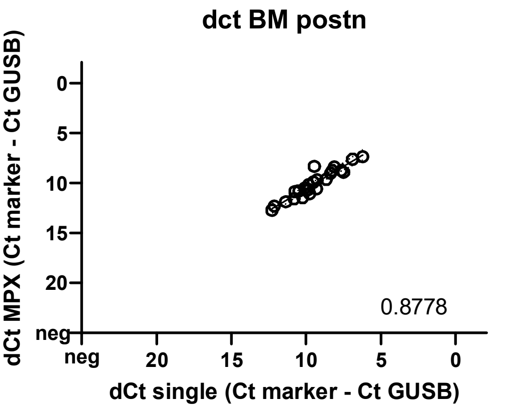
dct BM postn
dCt MPX (Ct marker - Ct GUSB)
dCt single (Ct marker - Ct GUSB)
0.8778

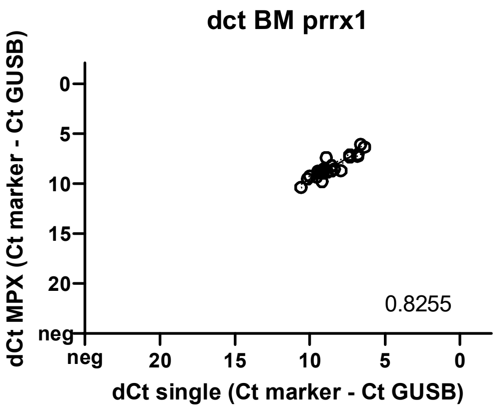
dct BM prrx1
dCt MPX (Ct marker - Ct GUSB)
dCt single (Ct marker - Ct GUSB)
0.8255

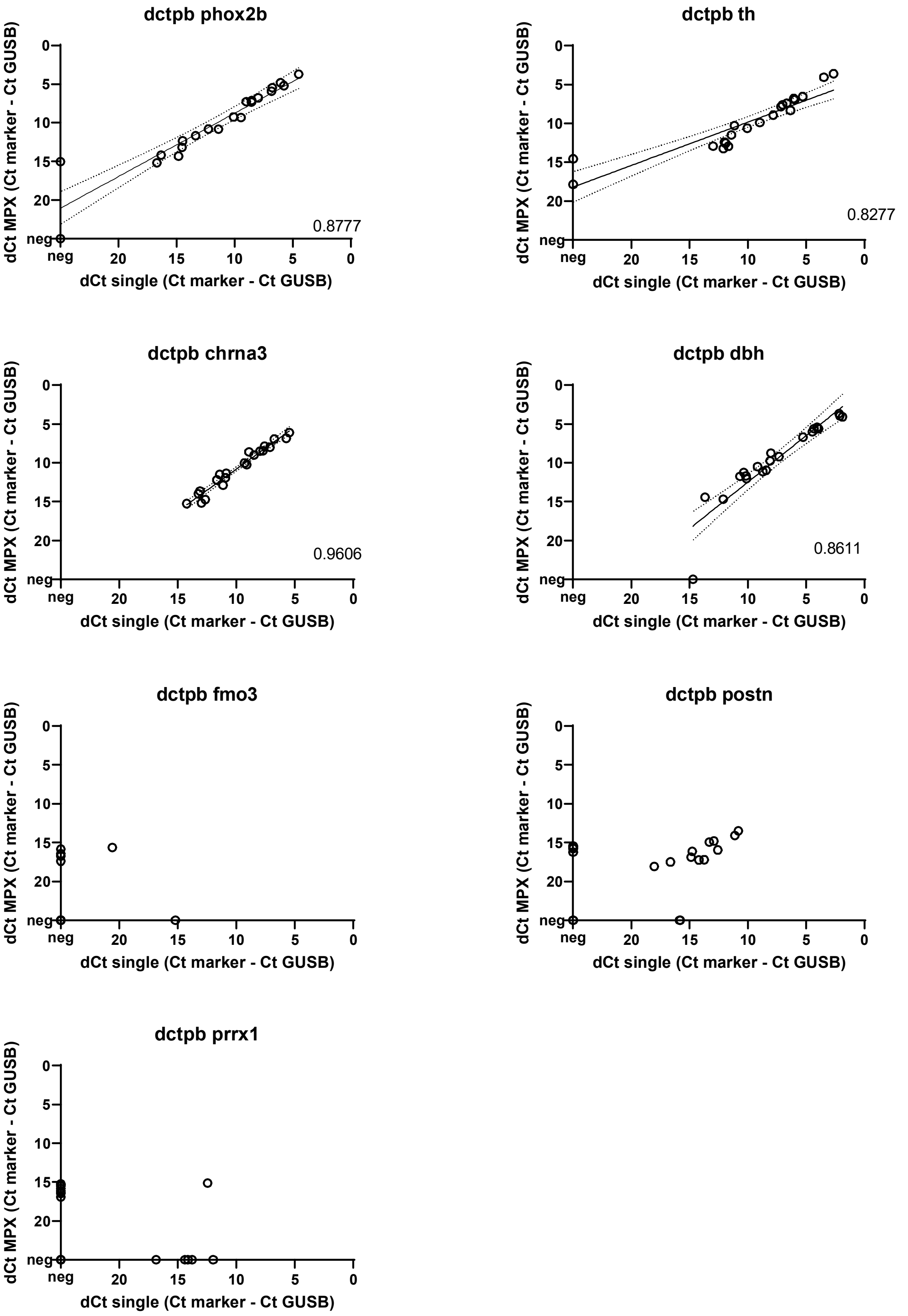

Figure 5. Correlation plots for individual samples per marker. (**A**) Bone marrow; (**B**)Peripheral blood

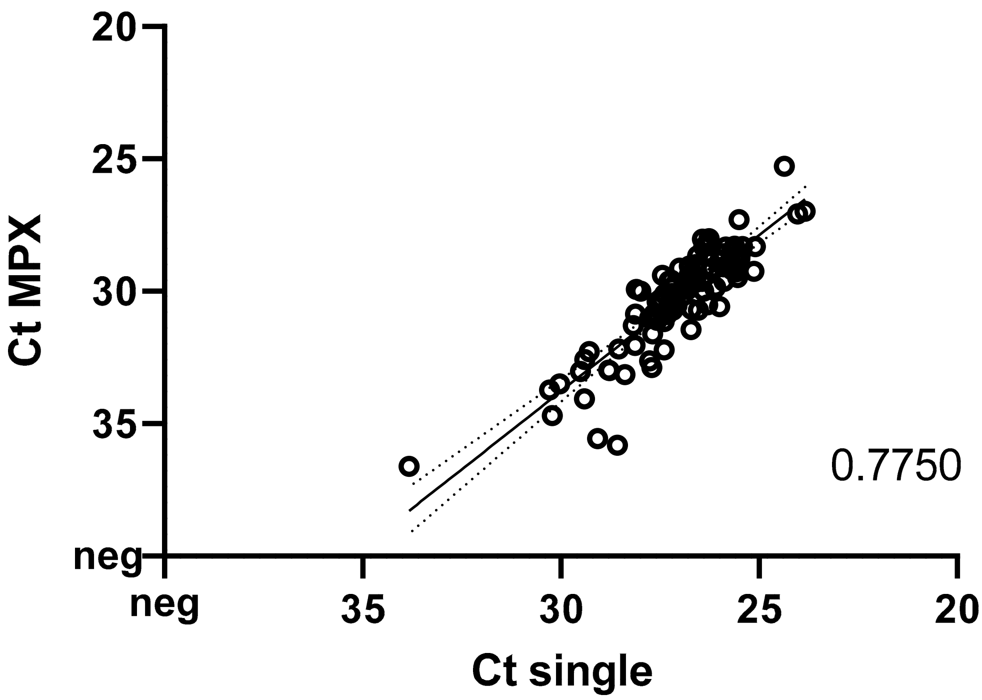

Figure S6. Correlation plot for FMO3 in MPX and singleplex in 100 BM samples.

Table 1. Primers and probe sequences for multiplex method.

Gene		Primer and Probe Sequence
TH	Forward primer	ATTGCTGAGATCGCCTTCCA
	Reverse primer	AATCTCCTCGGCGGTGTACTC
	Probe	**FAM**-ACAGGCACGGCGACCCGATTC-**BHQ1**
CHRNA3	Forward primer	GTCCATGTCTCAGCTGGTGAAG
	Reverse primer	TTCCATTTCAGCTTGTAGTCATTCC
	Probe	**DFO**-CAGATCATGGAGACCAACCTGTGGCTC-**BHQ2**
GAP43	Forward primer	CAAACCAGAAGATAAAGCTCATAAGGCCGC
	Reverse primer	GCTGTGCTGTATGAGAAGAACCA
	Probe	**YY**-ACGGAAGCTAGCCTGAATTTTG-**BHQ1**
DBH	Forward primer	TCCAGGGTCCTCCAGATATCTC
	Reverse primer	TGAGGAGTCGTTTCGTCCTCCT
	Probe	**YY-**CACCAGTGGGTTGTGGTAGTGAACTTCCAG-**BHQ1**
FMO3	Forward primer	GAATTCGGGCTGTGATATTGC
	Reverse primer	TCATCACCCAGGAGCCACTT
	Probe	**FAM**-TCAGCCGCACAGCAGAACAGGTCA-**BHQ1**
PRRX1	Forward primer	CCTGATGCTTTTGTGCGAGAA
	Reverse primer	TTTATTGGCTAGCATGGCTCTCT
	Probe	**DFO**-CAGAACCGAAGAGCCAAGTTCCGCA-**BHQ2**
POSTN	Forward primer	TTATATGAGAATGGAAGGAATGAAAGG
	Reverse primer	CGATCTCCTCCCTCAGTTTTGA
	Probe	**YY**-TGGGAGCCACCACAACGCAGC-**BHQ1**

Table S2. Examples Ct, ΔCt, AMPSCORE, CQCONF score and ΔRn results of MPX RT-qPCR, RNA qPCR and singleplex RT-qPCR.

Sample	MPX RT-qPCR					RNA (no RT) qPCR				Singpleplex RT-qPCR				
	Ct	ΔCt	AMPSCORE	CQCONF	ΔRn	CT	AMPSCORE	CQCONF	ΔRn	Ct	ΔCt	AMPSCORE	CQCONF	ΔRn
18-1372	30.00	11.45	0.85	0.87	0.29	30.92	0.83	0.88	0.36	UND	17.66	0.00	0.00	0.00
18-1372	29.81		1.19	0.31	1.62	31.14	0.83	0.87	0.32	37.03		0.83	0.58	0.21
18-1372	30.09		0.87	0.88	0.42					UND		0.00	0.00	0.00
18-2260	31.31	14.19	0.89	0.88	0.37	32.87	0.84	0.88	0.32	UND	18.19	0.00	0.00	0.00
18-2260	31.41		1.29	0.38	1.92	33.04	0.85	0.88	0.34	36.58		0.93	0.69	0.33
18-2260	31.44		0.91	0.86	0.32					UND		0.00	0.00	0.00
18-0159	30.65	12.07	1.37	0.39	2.88	32.07	0.82	0.84	0.29	32.85	13.47	1.29	0.87	1.88
18-0159	30.34		1.40	0.52	2.98	32.08	0.83	0.89	0.32	32.92		1.25	0.94	1.50
18-0159	30.05		1.42	0.52	3.19					34.36		1.22	0.91	1.29
18-0160	32.63	13.95	1.39	0.48	3.12	34.45	0.85	0.87	0.35	34.32	14.86	1.30	0.93	1.92
18-0160	32.28		1.38	0.62	2.79	34.25	0.85	0.91	0.35	34.58		1.25	0.92	1.40
18-0160	31.41		1.46	0.50	4.03					35.17		1.27	0.93	1.66

For the 4 examples from Supplemental Figure 2, the Ct value, ΔCt (Ct_{marker} – Ct_{GUSB}), AMPSCORE, CQCONF and ΔRn in MPX RT-qPCR, RNA qPCR (without RT) and singleplex RT-qPCR are shown, in triplicate. An AMPSCORE > 1 and CQCONF score > 0.5 are recommended. In the selected samples at least one of the replicates has an AMP score > 1 and ΔRn > 1, but one of the replicates has a CQCONF score < 0.5. For the singleplex RT-qPCR, samples 18-1372 and 18-2260 have a poor AMPSCORE and ΔRn < 1, suggesting these are false amplifications.

Table S3. Comparison of ΔCt and Ct results between multiplex and singleplex in control tissue.

Marker	BM				PB			
	Positive BM Samples Multiplex	Positive BM Samples Singleplex	Expression Multiplex (Mean)	Expression Singleplex (Mean)	positive PB Samples Multiplex	Positive PB Samples Singleplex	Expression Multiplex (Mean)	Expression Singleplex (Mean)
PHOX2B	0/54	0/51			0/50	0/37		
TH	17/54	15/51	16.1	15.3	21/50	10/37	14.1	15.2
CHRNA3	42/54	31/51	16.2	15.6	21/50	7/37	16.6	14.7
GAP43	42/54	20/51	16.8	14.9				
DBH					6/50	1/37	16.3	18.0
FMO3	54/54	48/48	8.6	10.8	16/50	8/104	14.8	15.5
PRRX1	54/54	46/48	10.4	11.8	30/50	43/104	15.2	14.7
POSTN	54/54	47/48	12.2	10.1	27/50	37/104	15.4	14.9
GUSB	54/54	99/99	19.9	22.7	50/50	141/141	21.6	23.6

All samples represent the mean of normalized Ct values ($\Delta Ct = Ct_{marker} - Ct_{GUSB}$), except *GUSB*, for which the mean Ct is reported. BM = bone marrow, PB = peripheral blood.

Table S4. Sample and patient characteristics of the cohort used to compare singleplex and multiplex RT-qPCR.

**Bone Marrow Samples**		
**Patient Number**	**Stage**	**Moment**
2012	M	After 3× N5N6
2013	M	After 1× N5N6
2014	M	After 1× N5N6
2015	MS	Progressive disease
2016	M	Relapse therapy
2024	M	After 1× N5N6
2029	M	After 1× N5N6
2031	M	After 1× N5N6
2031	M	Diagnosis
2033	M	Diagnosis
2043	M	Relapse therapy
2043	M	Relapse
2046	M	Diagnosis
2048	M	Diagnosis
2049	M	Diagnosis
2050	M	Diagnosis
2051	M	Relapse
2052	M	Diagnosis
2053	L2	Diagnosis
2056	M	After 2× N5N6 and 2× N8
2061	MS	Diagnosis
2071	M	After 1× N5N6
2072	M	Diagnosis
2081	M	Diagnosis
**Blood Samples**		
**Patient Number**	**Stage**	**Moment**
621	M	Relapse therapy
764	M	Relapse
772	M	Relapse
802	M	Relapse therapy
2015	MS	Progressive disease
2016	M	Relapse therapy
2016	M	Relapse
2024	M	Diagnosis
2033	M	Diagnosis
2050	M	Diagnosis
2063	M	Diagnosis
2074	M	Diagnosis
2080	L2	Diagnosis
2084	M	Diagnosis
G1	M	Diagnosis
G2	MS	Diagnosis
G3	M	Diagnosis
G4	M	Diagnosis
G5	M	Diagnosis
G6	M	Diagnosis
G7	M	Diagnosis

Stage according to International Neuroblastoma Risk Group Staging System. N5, N6 and N8 are cycles of induction chemotherapy. All patients were treated in accordance with the German NB2004 or Dutch NBL2009 trial for high-risk neuroblastoma [2,3].

Table S5. Detailed results of the comparison between MPX and singleplex RT-qPCR in a cohort of 24 bone marrow and 21 blood samples.

	**Bone Marrow**				**Peripheral Blood**		
PHOX2B	Singleplex +	Singleplex −	Total	PHOX2B	Singleplex +	Singleplex −	Total
MPX+	24	0	24	MPX+	19	1	20
MPX−	0	0	0	MPX−	0	1	1
Total	24	0	24	Total	19	2	21
TH-UK	Singleplex +	Singleplex −	Total	TH-UK	Singleplex +	Singleplex −	Total
MPX+	22	0	22	MPX+	13	0	13
MPX−	0	2	2	MPX−	5	3	8
Total	22	2	24	Total	18	3	21
CHRNA3	Singleplex +	Singleplex −	Total	CHRNA3	Singleplex +	Singleplex −	Total
MPX+	19	1	20	MPX+	16	0	16
MPX−	1	3	4	MPX−	0	5	5
Total	20	4	24	Total	16	5	21
GAP43	Singleplex +	Singleplex −	Total	DBH	Singleplex +	Singleplex −	Total
MPX+	18	6	24	MPX+	18	0	18
MPX−	0	0	0	MPX−	3	0	3
Total	18	6	24	Total	21	0	21
ADRN PANEL	Singleplex +	Singleplex −	Total	ADRN PANEL	Singleplex +	Singleplex −	Total
MPX+	24	0	24	MPX+	20	0	20
MPX−	0	0	0	MPX−	1	0	1
Total	24	0	24	Total	21	0	21
POSTN	Singleplex +	Singleplex −	Total	POSTN	Singleplex +	Singleplex −	Total
MPX+	6	1	7	MPX+	0	0	0
MPX−	0	17	17	MPX−	0	21	21
Total	6	18	24	Total	0	21	21
PRRX1	Singleplex +	Singleplex −	Total	PRRX1	Singleplex +	Singleplex −	Total
MPX+	6	3	9	MPX+	0	0	0
MPX−	3	12	15	MPX−	0	21	21
Total	9	15	24	Total	0	21	21
MES PANEL	Singleplex +	Singleplex −	Total	MES PANEL	Singleplex +	Singleplex −	Total
MPX+	8	3	11	MPX+	0	0	0
MPX−	2	11	13	MPX−	0	21	21

Total	10	14	24	Total	0	21	21

References

1. van Wezel, E.M.; Zwijnenburg, D.; Zappeij-Kannegieter, L.; Bus, E.; van Noesel, M.M.; Molenaar, J.J.; Versteeg, R.; Fiocco, M.; Caron, H.N.; van der Schoot, C.E.; et al. Whole-genome sequencing identifies patient-specific DNA minimal residual disease markers in neuroblastoma. *J. Mol. Diagn.: JMD* **2015**, *17*, 43–52.
2. Simon, T.; Berthold, F.; Borkhardt, A.; Kremens, B.; De Carolis, B.; Hero, B. Treatment and outcomes of patients with relapsed, high-risk neuroblastoma: Results of german trials. *Pediatric blood & cancer* **2011**, *56*, 578–583.
3. Kraal, K.C.; Bleeker, G.M.; van Eck-Smit, B.L.; van Eijkelenburg, N.K.; Berthold, F.; van Noesel, M.M.; Caron, H.N.; Tytgat, G.A. Feasibility, toxicity and response of upfront metaiodobenzylguanidine therapy therapy followed by german pediatric oncology group neuroblastoma 2004 protocol in newly diagnosed stage 4 neuroblastoma patients. *Eur. J. cancer* **2017**, *76*, 188–196.

© 2020 by the authors. Licensee MDPI, Basel, Switzerland. This article is an open access article distributed under the terms and conditions of the Creative Commons Attribution (CC BY) license (http://creativecommons.org/licenses/by/4.0/).
